# Targeting CDK8 dually enhances paclitaxel antitumor efficacy and alleviates chemotherapy-induced peripheral neuropathy in breast cancer

**DOI:** 10.3389/fphar.2026.1849893

**Published:** 2026-07-13

**Authors:** Xuejiao Hong, Dong Fang

**Affiliations:** 1 Department of Pharmacy, Henan Provincial People’s Hospital, Zhengzhou, China; 2 Institute of Chemical Biology, School of Pharmacy, Henan University, Kaifeng, China

**Keywords:** Cdk8, chemoresistance, paclitaxel, peripheral neuropathy, romaciclib

## Abstract

**Background:**

Paclitaxel-based chemotherapy remains a mainstay for breast cancer treatment, yet its efficacy is often limited by drug resistance and dose-limiting peripheral neuropathy. This study aimed to investigate whether CDK8 could serve as a dual target for enhancing the antitumor efficacy of paclitaxel while alleviating chemotherapy-induced neuropathic pain.

**Methods:**

We evaluated CDK8 expression in human breast cancer specimens before and after paclitaxel-containing neoadjuvant chemotherapy, as well as in paclitaxel-treated breast cancer cell lines and DRGs from syngeneic tumor-bearing mice. Loss- and gain-of-function approaches were performed to assess the functional role of CDK8 in tumor growth, paclitaxel sensitivity, and neuropathic pain behaviors. Myelin damage in the sciatic nerve was evaluated by transmission electron microscopy via G-ratio quantification. Finally, the selective CDK8/CDK19 inhibitor romaciclib was tested for its ability to enhance paclitaxel antitumor efficacy and alleviate paclitaxel induced peripheral neuropathy in a 4T1 murine breast cancer model.

**Results:**

CDK8 expression was markedly upregulated in human post-chemotherapy breast cancer tissues, paclitaxel-treated breast cancer cells, and DRGs of tumor-bearing mice. CDK8 overexpression promoted tumor growth and paclitaxel resistance, while CDK8 knockdown sensitized tumors to chemotherapy. In the peripheral nervous system, elevated CDK8 in DRGs recapitulated paclitaxel-induced mechanical allodynia, thermal hyperalgesia, and myelin damage, whereas CDK8 depletion prevented these neurotoxic effects. Pharmacological inhibition of CDK8 with romaciclib not only enhanced the antitumor efficacy of paclitaxel but also significantly alleviated paclitaxel-induced peripheral neuropathy *in vivo*.

**Conclusion:**

CDK8 is a dual target linking chemoresistance and neurotoxicity. Inhibiting CDK8 with romaciclib may improve paclitaxel therapy while reducing its side effects.

## 1 Introduction

Breast cancer is the most commonly diagnosed malignancy among women globally (Kimta et al., 2026). Paclitaxel-based chemotherapy remains a key treatment modality for this disease (Abouzeid et al., 2025). However, resistance to paclitaxel represents a major clinic challenge (Roskoski, 2024; Maghrebi-Ghojogh et al., 2025). Therefore, a better understanding of the mechanisms underlying paclitaxel resistance may facilitate the development of more effective preventive and therapeutic strategies.

In addition, paclitaxel-induced peripheral neuropathy is a dose-limiting adverse effect of chemotherapy, characterized by neuropathic pain, numbness, and tingling sensations in the hands and feet (Velasco-González and Coffeen, 2022). These sensory disturbances often severely impair the quality of life of cancer patients, leading to dose reductions or even discontinuation of anticancer treatment (Sawa et al., 2025; Burgess et al., 2021). Unfortunately, there are currently no effective interventions for preventing or managing chemotherapy-induced peripheral neuropathy. As such, identifying novel therapeutic targets for paclitaxel-induced peripheral neuropathy is urgently needed.

Cyclin-dependent kinase 8 (CDK8) is a member of the CDK family and belongs to the serine/threonine protein kinase group (Xie et al., 2026). Overexpression of CDK8 has been observed in various human cancers, including colon, cervical, and prostate cancers (Zhou et al., 2018; Li et al., 2024). High CDK8 expression is associated with shorter survival and poorer clinical outcomes in cancer patients, suggesting that it functions as an important oncogenic factor (Rzymski et al., 2015). Notably, knockdown of CDK8 has been shown to sensitize tumor cells to cisplatin in non-small cell lung cancer (NSCLC), indicating that CDK8 may represent a promising target for enhancing chemosensitivity (Zhang et al., 2014). Interestingly, CDK8 has also been reported to drive neurogenesis from a mesodermal lineage (Luo and Horvitz, 2017); however, whether CDK8 is involved in paclitaxel-induced peripheral neuropathy remains unclear. Therefore, this study aimed to investigate whether CDK8 could serve as a dual target for enhancing the antitumor efficacy of paclitaxel while alleviating chemotherapy-induced neuropathic pain in breast cancer treatment.

## 2 Materials and methods

### 2.1 Reagents and antibodies

Paclitaxel (580555) was purchased from Sigma-Aldrich (St. Louis, MO, United States). Romaciclib (HY-111388A) was purchased from MedChem Express (Monmouth Junction, NJ, United States). A rabbit-derived polyclonal antibody targeting CDK8 (ab272879) was obtained from Abcam (Cambridge, MA, United States), and was applied at a dilution of 1:1,000 for Western blotting and 1:200 for immunohistochemical staining. Horseradish peroxidase-conjugated secondary antibodies, including goat anti-rabbit IgG (ZB-2301) and goat anti-mouse IgG (ZB-2305), were acquired from Zhongshan Golden Bridge Company (Beijing, China), both used at a 1:1,000 dilution for Western blot analysis.

### 2.2 Cell lines and culture conditions

Human breast cancer cell lines MCF-7 and MDA-MB-231, along with the murine breast cancer cell line 4T1, were obtained from the Cell Bank of the Chinese Academy of Sciences (Shanghai, China). All cell lines were authenticated by short tandem repeat (STR) profiling using the PowerPlex 16HS System. Cells were maintained in RPMI 1640 medium supplemented with 10% fetal bovine serum (HyClone) and cultured at 37 °C in a humidified atmosphere with 5% CO_2_.

### 2.3 Plasmid construction, lentivirus packaging, and transduction

For CDK8 overexpression, the full-length CDK8 coding sequence was subcloned into the pCDH-CMV-MCS-EF1-CopGFP-T2A-Puro backbone. For CDK8 knockdown, oligonucleotides encoding CDK8-specific short hairpin RNA (shRNA) were inserted into the pLKO.1-puro vector. Lentiviral particles were generated by co-transfecting 293T cells with the packaging plasmid psPAX2 and the envelope plasmid pMD2.G. Viral supernatants were harvested at 24 and 48 h post-transfection, then clarified by filtration through 0.45 μm filters. The resulting lentiviral preparations were subsequently used to transduce breast cancer cells *in vitro* or to deliver shRNA into dorsal root ganglia (DRGs) in mice.

### 2.4 Human tissue specimens

A total of nine paired breast cancer tissue samples were collected from patients who underwent neoadjuvant chemotherapy containing paclitaxel at the First Hospital of Henan University between 2021 and 2024. Pre-treatment specimens were obtained via core needle biopsy at the time of initial diagnosis, while post-treatment specimens were surgically resected after the completion of paclitaxel-based neoadjuvant therapy. The nine patients with stage I to III breast cancer, ranging in age from 51 to 73 years, were enrolled. Their neoadjuvant chemotherapy regimens varied by subtype: two HR+/HER2- cases received TAC (paclitaxel, adriamycin, cyclophosphamide); four HER2+ cases were treated with THP (paclitaxel, trastuzumab, pertuzumab); and three triple negative breast cancer (TNBC) cases were given TC (paclitaxel, cyclophosphamide). All study protocols were reviewed and approved by the Ethics Committee of Henan University, and written informed consent was obtained from each patient prior to enrollment.

### 2.5 Animals and experimental procedures

Female BALB/c mice, aged 5–6 weeks, were obtained from Beijing Weitong Lihua Animal Technology Co., Ltd. All animal experiments were approved by the Institutional Animal Care and Use Committee of Henan University (approval No. HUSOM 2023-687). Animals were maintained under specific pathogen-free conditions with a 12-h light/dark cycle and provided with sterilized food and water *ad libitum*.

To assess the effect of paclitaxel on CDK8 expression, 4T1 murine breast cancer cells (5 × 10^6^ cells suspended in 100 μL of PBS) were implanted subcutaneously into the left flank of each mouse. One week after inoculation, animals received intraperitoneal injections of either paclitaxel (8 mg/kg) or vehicle control (Cremophor EL) every other day for a total of 2 weeks. This dosing regimen was selected based on the well-established dose-dependent nature of paclitaxel-induced peripheral neuropathy, and has been successfully employed in previous studies to induce a robust and reproducible neuropathic pain phenotype in mice (Shi et al., 2024). Following the treatment period, mice were euthanized by cervical dislocation, and tumor tissues along with L4-L5 dorsal root ganglia (DRGs) were harvested for analysis of CDK8 expression.

To investigate the functional role of CDK8 in tumor growth, 4T1 cells stably overexpressing CDK8 or carrying an empty vector control were injected subcutaneously (5 × 10^6^ cells in 100 μL PBS) into the left flank of BALB/c mice, and tumors were allowed to develop for 2 weeks. To examine whether CDK8 influences the response to paclitaxel, mice bearing established tumors from 4T1 cells stably transduced with CDK8 shRNA or control shRNA were treated 1 week after cell implantation with paclitaxel (8 mg/kg) or vehicle via intraperitoneal injection every other day for 2 weeks. Tumor tissues were subsequently collected for immunohistochemical analysis.

To evaluate the combined antitumor activity of paclitaxel and Romaciclib, 4T1 cells (5 × 10^6^ cells) were inoculated subcutaneously into the left flank of BALB/c mice. One week later, animals were randomly assigned to treatment groups using a computer-generated random number sequence and received intraperitoneal injections of vehicle, paclitaxel alone (8 mg/kg), Romaciclib alone (30 mg/kg), or the combination of both agents, administered every other day for 2 weeks. At the end of the treatment period, tumor weights were measured, and sciatic nerve specimens were collected for histopathological examination.

### 2.6 Intrathecal injection

Intrathecal drug delivery was performed using a 30-gauge needle attached to a 10 μL Hamilton syringe, following the method described by Hylden and Wilcox. Mice were anesthetized with 3% isoflurane delivered in oxygen, then firmly restrained by grasping the bilateral iliac crests. The L5 spinous process was identified by palpation, and the needle was inserted into the intervertebral space between L5 and L6. Correct needle placement was confirmed by a characteristic tail flick response. The drug solution was administered slowly over a period exceeding 20 s, and the needle was left in place for an additional 20 s before withdrawal.

### 2.7 Behavior testing

All behavioral testing was conducted by an investigator blinded to the experimental group assignments. To standardize testing conditions, the order of testing across different treatment groups was systematically rotated each day, and all assessments were performed between 9:00 a.m. and 2:00 p.m. to control for circadian influences. To assess mechanical sensitivity, mice were placed individually in transparent plastic enclosures positioned on a wire mesh platform and allowed to acclimate for at least 30 min prior to testing. A series of von Frey filaments (ranging from 0.16 to 2.0 g) were applied perpendicularly to the plantar surface of the right hind paw, with each filament maintained in contact for 6–8 s. Each stimulus strength was applied five times at intervals of 30 s, and the threshold was defined as the minimum force that elicited at least three withdrawal responses out of five applications.

Thermal sensitivity was evaluated using a radiant heat stimulator (BME410A; Institute of Biological Medicine, Academy of Medical Science, Tianjin, China) directed at the plantar surface of the right hind paw. The time elapsed between stimulus onset and paw withdrawal was recorded as the paw withdrawal latency (PWL). Each paw was tested three times with intervals of at least 5 min between trials, and the average latency was calculated. A cutoff time of 30 s was implemented to prevent potential tissue injury.

### 2.8 Histopathologic evaluation of the sciatic nerve

Sciatic nerve specimens were dissected from mice and fixed overnight at 4 °C in 2.5% glutaraldehyde. Following primary fixation, tissues were postfixed in 1% osmium tetroxide for 2 h, then dehydrated and embedded in Epon 812 epoxy resin (Electron Microscopy Sciences, Hatfield, PA, United States). Ultrathin sections of 90 nm thickness were cut, stained with uranyl acetate and lead citrate, and subsequently examined under a transmission electron microscope (Hitachi, Chiyoda City, Japan) to evaluate morphological alterations. Myelination integrity was quantified by calculating the G-ratio, defined as the ratio of the inner axonal diameter to the total outer fiber diameter (axon plus myelin sheath). For each treatment group, sciatic nerves were collected from three mice. From each animal, five randomly selected, non-overlapping fields containing myelinated axons were imaged at a consistent magnification. All clearly identifiable myelinated fibers within each field were analyzed, yielding approximately 15–20 individual fibers per mouse across experimental groups.

### 2.9 Immunohistochemistry (IHC)

Human breast cancer specimens, mouse tumor tissues, and L4-L5 dorsal root ganglia were fixed in 4% paraformaldehyde and embedded in paraffin. Tissue sections were cut at 4 μm, mounted onto polylysine-coated slides, and subjected to deparaffinization with xylene followed by rehydration through graded ethanol. For immunohistochemical staining, sections were incubated overnight at 4 °C with a primary antibody against CDK8 (diluted 1:100). After washing, sections were incubated with HRP-conjugated secondary antibody for 30 min, and immunoreactivity was visualized using diaminobenzidine (DAB). CDK8 expression was evaluated independently by two pathologists who were blinded to the experimental groups. Staining intensity was graded on a scale of 0–3 (0, negative; 1, weak; 2, moderate; 3, strong), while the proportion of positively stained cells was scored from 0 to 5 (0, no staining; 1, <1%; 2, 2%–10%; 3, 11%–30%; 4, 31%–70%; 5, 71%–100%). A final composite score (ranging from 0 to 8) was generated by summing the intensity and extent scores for each specimen, and the average score from the two pathologists was used for analysis.

### 2.10 Real-time PCR

Total RNA was extracted using TRIzol reagent (Invitrogen, Carlsbad, CA, United States), and RNA concentration and purity were assessed with a NanoDrop One spectrophotometer (Thermo Fisher Scientific, Waltham, MA, United States), with all samples exhibiting an A260/A280 ratio exceeding 1.8. For reverse transcription, 1 μg of total RNA was converted to complementary DNA (cDNA) using the PrimeScript RT Reagent Kit (Takara, Dalian, China). Quantitative PCR (qPCR) was performed on a MiniOpticon Real-Time PCR System (Bio-Rad, Hercules, CA, United States) using SYBR Green Master Mix (Applied Biosystems, Foster City, CA, United States). Gene expression levels were calculated using the 2^−ΔΔCt^ method and normalized to glyceraldehyde-3-phosphate dehydrogenase (GAPDH) as an internal reference. The primer sequences employed for human samples were as follows: CDK8 forward, 5′- GCT GAT AGG AAG GTG TGG CT-3′ and reverse, 5′- GCA AAG CCC ATG TCA GCA AT-3′; GAPDH forward, 5′-GAC ACC CAC TCC TCC ACC TTT-3′ and reverse, 5′-TTG CTG TAG CCA AAT TCG TTG T-3′. For mouse tissues, the primer sets were: CDK8 forward, 5′- GAA GGT CCT GAG CGA GGA AG-3′ and reverse, 5′- GGT ATA ATG TCG CGC TCC GA-3′; GAPDH forward, 5′-ACC CAG AAG ACT GTG GAT GG-3′ and reverse, 5-CAC ATT GGG GGT AGG AAC AC-3′.

### 2.11 Protein extraction and western blotting

Tissue specimens and cultured cells were lysed using RIPA buffer (Beyotime Biotechnology, Shanghai, China) supplemented with 0.25 mM phenylmethylsulfonyl fluoride and a protease inhibitor cocktail. Protein concentrations were determined with a BCA Protein Assay Kit (Pierce, Rockford, IL, United States). Equal amounts of protein lysates were resolved by 10% SDS-polyacrylamide gel electrophoresis and subsequently transferred onto polyvinylidene difluoride (PVDF) membranes. Following transfer, membranes were blocked with 5% bovine serum albumin (BSA) dissolved in Tris-buffered saline containing 0.1% Tween-20 (TBST) for 1 h at ambient temperature, then incubated overnight at 4 °C with the indicated primary antibodies. After three washes with TBST, membranes were incubated with horseradish peroxidase (HRP)-conjugated secondary antibodies for 1 h at room temperature. Immunoreactive bands were visualized using enhanced chemiluminescence reagents and captured with a FluorChem E Imager System (Protein Simple, San Jose, CA, United States). Densitometric quantification was performed using Quantity One software (Bio-Rad, Hercules, CA, United States).

### 2.12 Statistical analysis

All quantitative data are presented as mean ± standard error of the mean (SEM). Statistical analyses were conducted using GraphPad Prism version 8.0 (GraphPad Software, La Jolla, CA, United States). Comparisons between two independent groups were performed using unpaired Student’s t-test. For comparisons involving more than two groups, one-way analysis of variance (ANOVA) followed by Dunnett’s *post hoc* test was applied, whereas two-way ANOVA with Bonferroni’s correction was used for analyses involving two independent variables. A significance threshold of p < 0.05 was adopted for all statistical tests.

## 3 Results

### 3.1 Paclitaxel upregulates CDK8 expression in breast cancer

**FIGURE 1:**
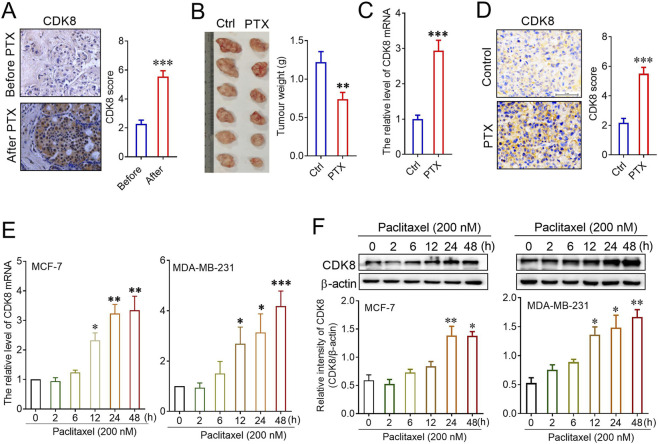
Paclitaxel induces CDK8 upregulation both *in vivo* and in breast cancer cell lines. **(A)** Immunohistochemical staining showing CDK8 levels in human breast cancer specimens before and after paclitaxel-containing neoadjuvant chemotherapy. ***p < 0.001, two-tailed paired t-test, n = 9 patients per group. **(B)** Effects of paclitaxel on tumor growth in a mouse syngeneic tumor model treated with paclitaxel (8 mg/kg every other day for 2 weeks). **p < 0.01, a two-tailed unpaired t-test, n = 6 mice per group. **(C)** Quantitative real-time PCR analysis of CDK8 mRNA levels in mouse tumor tissues following paclitaxel treatment. ***p < 0.01, two-tailed unpaired t-test, n = 6 mice per group. **(D)** Immunohistochemical analysis of CDK8 protein expression in mouse tumor tissues after paclitaxel treatment. ***p < 0.01, two-tailed unpaired t-test, n = 6 mice per group. **(E,F)** CDK8 mRNA **(E)** and protein **(F)** expression levels in MCF-7 and MDA-MB-231 cells following exposure to 200 nM paclitaxel. *p < 0.05, **p < 0.01, ***p < 0.001, one-way ANOVA, n = 3 independent experiments per group.

To explore whether CDK8 may serve as a viable target for increasing paclitaxel sensitivity in breast cancer, we first evaluated its expression in nine human breast cancer specimens obtained prior to and following neoadjuvant chemotherapy that included paclitaxel. Immunohistochemical analysis demonstrated a pronounced increase in CDK8 levels in tumor tissues after treatment when compared with samples collected before treatment (Figure 1A). Because these patients received additional chemotherapeutic agents alongside paclitaxel, we subsequently utilized a mouse syngeneic tumor model established with 4T1 murine breast cancer cells to assess the effect of paclitaxel alone on both tumor growth and CDK8 expression. Despite observing a delay in tumor progression following paclitaxel administration at a dose of 8 mg/kg every other day for 2 weeks (Figure 1B), we found that paclitaxel treatment led to elevated CDK8 mRNA and protein expression within the tumor tissues (Figures 1C,D).

To confirm whether paclitaxel directly induces CDK8 expression in breast cancer cells, we exposed MCF-7 and MDA-MB-231 cells with 200 nM paclitaxel and examined CDK8 expression. Consistent with the *in vivo* observations, paclitaxel treatment resulted in increased CDK8 mRNA and protein expression across all four cell lines (Figures 1E,F). Taken together, these findings suggest that paclitaxel enhances CDK8 expression in breast cancer cells.

### 3.2 CDK8 serves as a potential target for enhancing paclitaxel sensitivity in breast cancer

**FIGURE 2:**
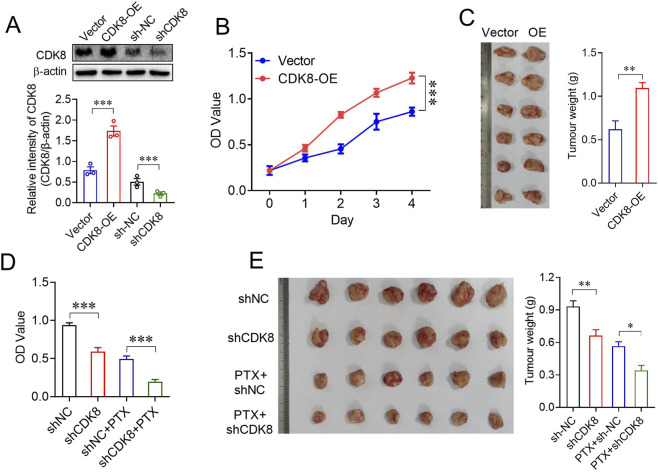
CDK8 suppresses paclitaxel efficacy in breast cancer. **(A)** Validation of CDK8 protein levels in 4T1 cells stably engineered for CDK8 overexpression or knockdown via Western blotting. ***p < 0.001, one-way ANOVA, n = 3 independent experiments per group. **(B)** Proliferation curves of control and CDK8-overexpressing 4T1 cells determined by MTT assays. ***p < 0.001, two-way ANOVA, n = 5 independent experiments per group. **(C)** *In vivo* tumor growth following subcutaneous implantation of CDK8-overexpressing versus control 4T1 cells into BALB/c mice. **p < 0.01, two-tailed unpaired t-test, n = 6 mice per group. **(D)** MTT assessment of paclitaxel sensitivity in CDK8-knockdown 4T1 cells compared with controls. ***p < 0.001, two-way ANOVA, n = 5 independent experiments per group. **(E)** *In vivo* evaluation of paclitaxel response using syngeneic tumor models derived from CDK8-knockdown or control 4T1 cells. Mice were treated with paclitaxel (8 mg/kg, intraperitoneal injection every other day for 2 weeks) starting 1 week post-inoculation. sh-NC: vector control; shCDK8: CDK8 knockdown; sh-NC + PTX: vector control with paclitaxel; shCDK8+PTX: CDK8 knockdown with paclitaxel. *p < 0.05, **p < 0.01, one-way ANOVA, n = 6 mice per group.

To explore whether the upregulation of CDK8 induced by paclitaxel may compromise chemotherapeutic efficacy in breast cancer, we first generated 4T1 cell lines with stable CDK8 overexpression and stable CDK8 knockdown. Western blot analysis confirmed successful construction of these cell lines (Figure 2A). The effect of CDK8 on tumor cell proliferation was assessed using MTT assays. Results demonstrated that CDK8 overexpression significantly promoted cell growth (Figure 2B). In parallel, subcutaneous inoculation of CDK8-overexpressing 4T1 cells into BALB/c mice resulted in markedly accelerated tumor growth compared with control cells (Figure 2C).

To further investigate the role of CDK8 in modulating paclitaxel sensitivity, we incubated CDK8-knockdown 4T1 cells with 200 nM paclitaxel for 48 h. MTT assays revealed that CDK8 knockdown significantly enhanced the inhibitory effect of paclitaxel on tumor cell viability (Figure 2D). Concurrently, we established syngeneic tumor mouse models using 4T1 cells stably transfected with CDK8 knockdown or vector control constructs. One week after tumor inoculation, mice received intraperitoneal injections of paclitaxel (8 mg/kg) or vehicle every other day for a duration of 2 weeks. Notably, CDK8 knockdown substantially enhanced the antitumor efficacy of paclitaxel *in vivo* (Figure 2E). Collectively, these findings suggest that CDK8 represents a promising target for sensitizing breast cancer to paclitaxel-based therapy.

### 3.3 CDK8 plays a role in paclitaxel-induced peripheral neuropathy

**FIGURE 3:**
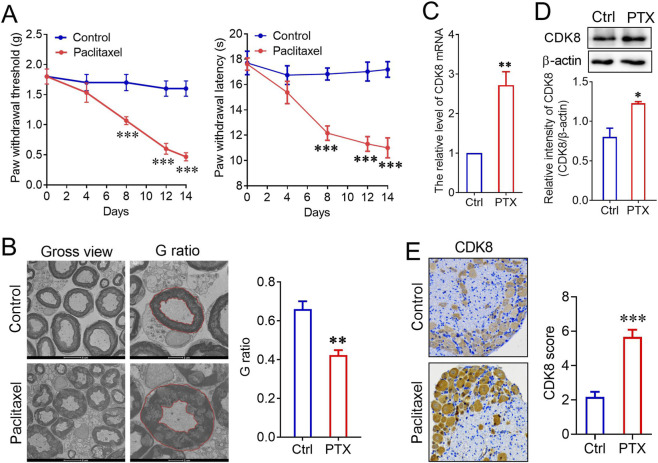
Paclitaxel triggers peripheral neuropathy and upregulates CDK8 in dorsal root ganglia of syngeneic tumor-bearing mice. **(A)** Assessment of mechanical allodynia (left) and thermal hyperalgesia (right) in syngeneic tumor-bearing mice following paclitaxel administration. ***p < 0.001, two-way ANOVA, n = 6 mice per group. **(B)** Representative transmission electron microscopy images of sciatic nerve sections and quantitative analysis of G-ratios in myelinated fibers. *p < 0.05, two-tailed unpaired t-test, n = 3 mice per group. **(C,D)** CDK8 mRNA **(C)** and protein **(D)** levels measured in L4-L5 dorsal root ganglia (DRG) from syngeneic tumor-bearing mice treated with paclitaxel versus vehicle. *p < 0.05, **p < 0.01, two-tailed unpaired t-test, n = 3 mice per group. **(E)** Immunohistochemical staining showing CDK8 expression in L4-L5 DRG sections from vehicle-treated (Ctrl) and paclitaxel-treated (PTX) syngeneic tumor-bearing mice. ***p < 0.001, two-tailed unpaired t-test, n = 6 mice per group.

Paclitaxel-induced peripheral neuropathy represents a serious complication that profoundly affects the quality of life of patients. In line with previous studies, our experiments demonstrated that administration of paclitaxel in syngeneic tumor-bearing mice led to the development of mechanical allodynia and thermal hyperalgesia (Figure 3A). Additionally, paclitaxel exposure resulted in myelin damage within the sciatic nerve, as evidenced by a marked reduction in the G-ratio of myelinated fibers (Figure 3B).

Sensory neurons located in the dorsal root ganglia (DRG) are particularly vulnerable to paclitaxel accumulation due to the presence of a fenestrated blood-DRG barrier with higher permeability (Wei et al., 2026). We therefore examined CDK8 expression in the DRGs of tumor-bearing mice following paclitaxel treatment. Quantitative real-time PCR and Western blot analyses revealed that paclitaxel significantly increased both CDK8 mRNA and protein levels in the L4-L5 DRGs compared with vehicle-treated controls (Figures 3C,D). Consistently, immunohistochemical staining of L4-L5 DRG sections further confirmed a marked elevation of CDK8 protein expression in paclitaxel-treated mice (Figure 3E). Collectively, these results indicate that paclitaxel not only induces peripheral neuropathy but also upregulates CDK8 expression in the DRG.

**FIGURE 4:**
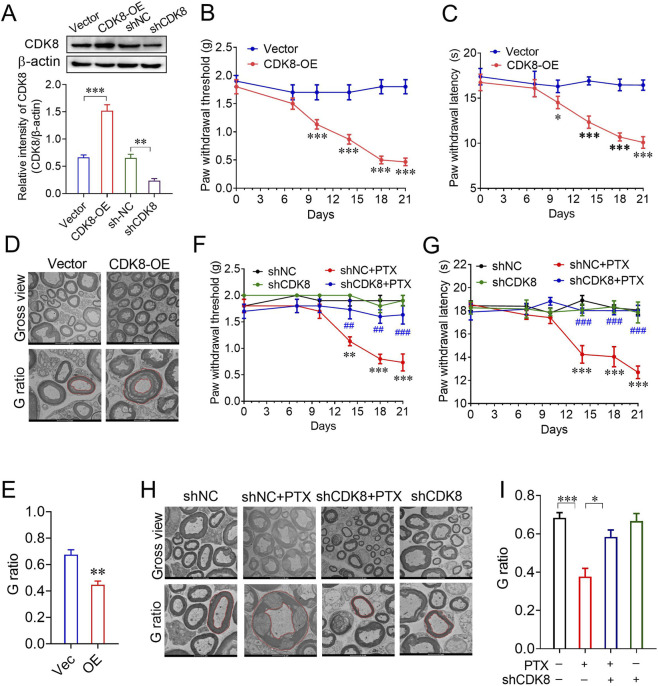
CDK8 mediates paclitaxel-induced peripheral neuropathy in mice. **(A)** Western blot verification of CDK8 protein levels in L4-L5 DRGs following stable overexpression or knockdown of CDK8. **p < 0.01, ***p < 0.001, one-way ANOVA, n = 3 independent experiments per group. **(B,C)** Behavioral assessments of mechanical allodynia **(B)** and thermal hyperalgesia **(C)** in normal mice overexpressing CDK8 compared with vector controls. *p < 0.05, ***p < 0.001, two-way ANOVA, n = 6 mice per group. **(D,E)** T Representative transmission electron microscopy images of sciatic nerve sections **(D)** and corresponding G-ratio quantification **(E)** from mice receiving vector control or CDK8 overexpression. **p < 0.01, two-tailed unpaired t-test, n = 4 mice per group. **(F,G)** Evaluation of paclitaxel-induced mechanical allodynia **(F)** and thermal hyperalgesia **(G)** in mice with or without CDK8 knockdown. **p < 0.01, ***p < 0.001 versus shNC group; ^##^p < 0.01, ^###^p < 0.001 versus PTX group; two-way ANOVA, n = 6 mice per group. **(H,I)** Transmission electron microscopy analysis of sciatic nerve morphology **(H)** and G-ratio quantification **(I)** across four experimental groups: shNC, shCDK8, shNC + PTX, and shCDK8+PTX. *p < 0.05, **p < 0.01, one-way ANOVA, n = 3 mice per group.

To determine whether CDK8 contributes to paclitaxel-induced peripheral neuropathy, we manipulated CDK8 expression in the L4-L5 DRGs of normal mice via viral delivery. Western blot analysis confirmed successful CDK8 overexpression and knockdown, respectively (Figure 4A). As expected, exogenous CDK8 expression resulted in significant reductions in both mechanical withdrawal threshold and thermal withdrawal latency (Figures 4B,C). Furthermore, CDK8 overexpression in the DRGs induced myelin damage and a decreased G-ratio in the sciatic nerve (Figures 4D,E).

Conversely, intrathecal delivery of CDK8-specific shRNA effectively knocked down CDK8 in DRGs. One week later, mice received paclitaxel (8 mg/kg) or vehicle every other day for 2 weeks. Notably, suppression of CDK8 almost completely abrogated paclitaxel-induced mechanical allodynia and thermal hyperalgesia (Figures 4F,G). In addition, CDK8 depletion mitigated paclitaxel-induced myelin damage in the sciatic nerve (Figures 4H,I). Together, these findings indicate that CDK8 plays a critical role in mediating paclitaxel-induced peripheral neuropathy, and that targeting CDK8 in DRGs may represent a potential strategy to mitigate this debilitating side effect.

### 3.4 Romaciclib enhances paclitaxel antitumor efficacy and mitigates paclitaxel-induced peripheral neuropathy *in vivo*

**FIGURE 5:**
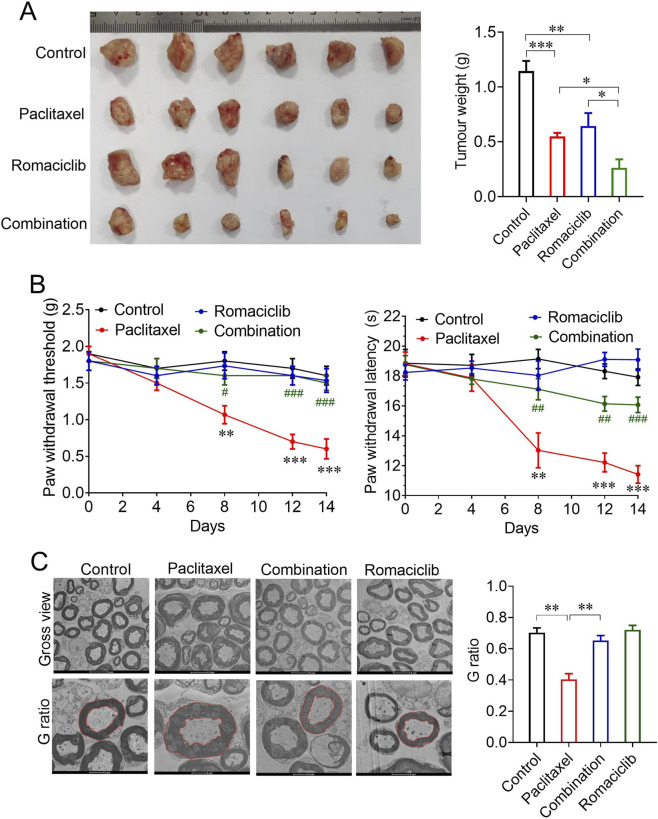
Romaciclib enhances the antitumor activity of paclitaxel and alleviates paclitaxel-induced peripheral neuropathy *in vivo*. **(A)** Antitumor effect of romaciclib in combination with paclitaxel in a 4T1 syngeneic tumor model. *p < 0.05, **p < 0.01, ***p < 0.001, one-way ANOVA, n = 6 mice per group. **(B)** Behavioral evaluation of paclitaxel-induced mechanical allodynia (left) and thermal hyperalgesia (right) following romaciclib co-administration. **p < 0.01, ***p < 0.001 versus control group; #p < 0.05, ##p < 0.01, ###p < 0.001 versus PTX group; two-way ANOVA, n = 6 mice per group. **(C)** Ultrastructural analysis of sciatic nerve sections via transmission electron microscopy and quantification of G-ratios across four treatment groups: vehicle, PTX alone, romaciclib alone, and PTX plus romaciclib (Combination). **p < 0.01, one-way ANOVA, n = 3 mice per group.

Given that paclitaxel treatment leads to upregulation of CDK8 expression, and considering that romaciclib, a selective CDK8/CDK19 inhibitor, has entered clinical trials for the treatment of acute myeloid leukemia, we next investigated whether this inhibitor could augment the antitumor activity of paclitaxel in breast cancer. To address this question, we administered romaciclib and/or paclitaxel via intraperitoneal injection to mice bearing syngeneic 4T1 tumors. As anticipated, the combination of paclitaxel with romaciclib resulted in markedly greater suppression of tumor growth compared with paclitaxel alone (Figure 5A).

We further explored whether romaciclib could ameliorate paclitaxel-induced nerve injury and neuropathic pain. Behavioral assessments revealed that administration of romaciclib significantly alleviated paclitaxel-induced mechanical allodynia and thermal hyperalgesia in the 4T1 syngeneic tumor model (Figure 5B). Ultrastructural examination of the sciatic nerve further demonstrated that Romaciclib markedly attenuated paclitaxel-induced myelin damage (Figure 5C). Collectively, these results indicate that romaciclib is capable of reducing the severity of paclitaxel-induced peripheral neuropathy *in vivo*.

## 4 Discussion

In this study, we identified CDK8 as a key factor linking paclitaxel treatment to both chemoresistance and peripheral neuropathy in breast cancer. Paclitaxel exposure upregulated CDK8 in breast cancer cells and DRGs. Within tumors, CDK8 promoted cell proliferation and paclitaxel resistance, whereas its knockdown sensitized tumors to chemotherapy. In the peripheral nervous system, elevated CDK8 in DRGs induced mechanical allodynia, thermal hyperalgesia, and myelin damage, recapitulating paclitaxel-induced neuropathy. Conversely, CDK8 depletion largely prevented these neurotoxic effects. Accordingly, pharmacological inhibition of CDK8 with romaciclib not only enhanced the antitumor efficacy of paclitaxel but also alleviated paclitaxel-induced peripheral neuropathy. Collectively, these findings position CDK8 as a dual-purpose therapeutic target that addresses two major clinical challenges associated with paclitaxel-based chemotherapy in breast cancer.

CDK8 is frequently overexpressed in a variety of human malignancies, including colorectal, breast, prostate, and glioma, and its elevated expression is generally associated with poor clinical outcomes (Firestein et al., 2008; Knab et al., 2021; Fukasawa et al., 2021). In this study, our results showed that paclitaxel treatment upregulates CDK8 expression in breast cancer cells, which in turn compromises the antitumor efficacy of paclitaxel. Although the precise mechanisms by which CDK8 counteracts paclitaxel sensitivity were not fully elucidated in the present study, several possibilities may be proposed based on existing literature. First, CDK8 is known to modulate the WNT/β-catenin pathway, a key signaling cascade involved in tumor progression and chemoresistance (Firestein et al., 2008; Cai et al., 2015). Second, CDK8 can phosphorylate STAT1 at serine 727, thereby suppressing natural killer (NK) cell activity and impairing antitumor immune responses (Putz et al., 2013). Third, CDK8 has been shown to maintain stemness and tumorigenicity through regulation of the c-MYC pathway, which could promote the survival of chemotherapy-resistant cancer cell subpopulations (Fukasawa et al., 2021; Wang et al., 2025).

Interestingly, these same pathways, particularly STAT1 and WNT/β-catenin, have also been implicated in peripheral neuropathy. Recent studies have shown that targeting STAT1 signaling modulates microglial polarization in diabetic peripheral neuropathy (Wei et al., 2026), and that inhibiting WNT/β-catenin signaling alleviates paclitaxel induced neuropathic pain (Resham and Sharma, 2019). These findings support our hypothesis that CDK8 may act through these pathways to regulate both chemoresistance and peripheral neuropathy. In addition, recent studies in osteoarthritis have shown that CDK8 promotes inflammatory responses through NF-κB mediated transcriptional regulation (Lin et al., 2025). These observations led us to investigate whether CDK8 also contributes to paclitaxel induced peripheral neuropathy (PIPN), a debilitating complication that affects a large proportion of breast cancer patients and manifesting as neuropathic pain, sensory loss, and motor dysfunction (Shi et al., 2024; Staff et al., 2020). Despite extensive investigations implicating aberrant ion channel activity, neurotransmitter dysregulation, and neuroinflammation in the pathogenesis of PIPN, effective preventive or therapeutic interventions remain lacking (Segura-Chama et al., 2026; Sharma et al., 2025; de la Peña et al., 2025). Our data demonstrate that paclitaxel upregulates CDK8 in mouse DRGs, and that CDK8 overexpression alone recapitulates neuropathic pain and myelin damage, whereas CDK8 knockdown or pharmacological inhibition alleviates these effects. Together, these findings position CDK8 as a shared regulator linking paclitaxel resistance and peripheral neuropathy, though the relative contributions of WNT/β-catenin, STAT1, c-MYC, and NF-κB pathways to each phenotype warrant further investigation.

CDK8 has garnered considerable attention as an anticancer drug target, yet selective CDK8 inhibitors have not advanced to clinical approval (Lin et al., 2024). Due to the high sequence homology between CDK8 and its paralog CDK19, most reported small-molecule inhibitors exhibit dual CDK8/19 inhibitory activity (Khamidullina et al., 2025; Xu et al., 2024). Among these, several dual inhibitors have entered clinical trials. For instance, Romaciclib (also known as SEL120-34A) has progressed to Phase II trials for the treatment of acute myeloid leukemia (AML), and Senexin B has completed Phase I evaluation in patients with advanced solid tumors (Rzymski et al., 2017; Ding et al., 2022). These clinical advancements underscore the therapeutic potential of targeting the CDK8/19 axis in oncology. Notably, while CDK8/19 inhibitors have primarily been investigated for their direct antitumor effects, our findings reveal an additional benefit of alleviating chemotherapy-induced neurotoxicity, a common dose-limiting side effect that often compromises treatment adherence and quality of life.

Despite these promising findings, several translational limitations of CDK8/19 inhibition warrant consideration. First, the chronic toxicity of sustained CDK8/19 inhibition, particularly in the context of peripheral nerve homeostasis, has not been evaluated. Since CDK8 is involved in neurogenesis (Luo and Horvitz, 2017). Second, most inhibitors including romaciclib also inhibit CDK19, whose distinct role in neuropathic pain has not been characterized. Third, the pharmacokinetic properties of romaciclib, including blood nerve barrier permeability, require further optimization. Given that romaciclib has shown favorable safety profiles in early phase studies, its combination with paclitaxel warrants further clinical evaluation. Such a combination strategy could simultaneously address chemoresistance and neurotoxicity in breast cancer, though rigorous translational research is still needed.

Several limitations of this study should be acknowledged. First, CDK8 overexpression by lentiviral transduction may exceed physiological levels observed in patients. Although our human IHC data confirmed elevated CDK8 after paclitaxel treatment, the semiquantitative nature of this analysis precludes direct comparison with our overexpression model. Future studies using genetic models with endogenous CDK8 upregulation are needed. Second, we did not identify which specific cell types within the DRG exhibit CDK8 upregulation following paclitaxel treatment, including sensory neurons, satellite glial cells, Schwann cells, or immune cells. Third, as romaciclib is a dual CDK8/CDK19 inhibitor, we cannot exclude a potential contribution from CDK19 inhibition. Future studies using cell type specific knockout models and selective CDK8 inhibitors are necessary. Fourth, intrathecal viral delivery may itself alter inflammatory signaling or affect multiple cell populations. However, our control groups did not show significant neuropathic pain behaviors or myelin damage, suggesting minimal off target effects. Finally, human breast cancer specimens were obtained from patients who received paclitaxel based neoadjuvant chemotherapy combined with other agents, so we cannot definitively attribute CDK8 upregulation to paclitaxel alone in clinical samples. Nevertheless, our mouse model and cell line experiments confirmed that paclitaxel alone is sufficient to induce CDK8 expression.

## Data Availability

The raw data supporting the conclusions of this article will be made available by the authors, without undue reservation.
